# Development of a Sensitive and Rapid Method for Determination of Acrylamide in Bread by LC-MS/MS and Analysis of Real Samples in Iran IR 

**DOI:** 10.22037/ijpr.2019.111994.13474

**Published:** 2020

**Authors:** Samira Eslamizad, Farzad Kobarfard, Kimia Tabib, Hassan Yazdanpanah, Jamshid Salamzadeh

**Affiliations:** a *Food Safety Research Center, Shahid Beheshti University of Medical Sciences, Tehran, Iran. *; b *Department of Medicinal Chemistry, School of Pharmacy, Shahid Beheshti University of Medical Sciences, Tehran, Iran. *; c *Phytochemistry Research Center, Shahid Beheshti University of Medical Sciences, Tehran, Iran. *; d *Department of Toxicology and Pharmacology, School of Pharmacy, Shahid Beheshti University of Medical Sciences, Tehran, Iran.*

**Keywords:** Food safety, Acrylamide, Method validation, Sangak bread, LC-MS/MS, Iran IR

## Abstract

A new sample preparation procedure and a liquid chromatography–tandem mass spectrometry (LC–MS/MS) method were developed for the quantitative analysis of acrylamide in bread. The method is based on sample extraction in methanol, purification with Carrez solutions and clean- up with Primary Secondary Amine (PSA).The developed method offers an efficient, inexpensive, easy sample preparation and very sensitive procedure for determination of acrylamide in bread. The use of spiked calibration curves for constructing the calibration curve substantially reduced adverse matrix-related effects. Recoveries were between 96 and 105.3%. Good results were obtained with respect to repeatability (RSDs <11%). The limit of detection and quantification of the method was 0.3 and 1 ng/g, respectively, which shows the method is very sensitive. The developed method was used for the determination of acrylamide in 26 traditional bread samples (Sangak) collected from Shiraz. The results showed that about 96% of Sangak bread samples were contaminated with acrylamide that 64.3 and 33.3 of semi-industrial and traditional Sangak bread were higher than benchmark levels (50 µg/kg), respectively. There are a few reports concerning contamination of Sangak bread samples with acrylamide in Iran. Therefore, this method could be used for a comprehensive survey of acrylamide in Sangak bread samples in the country.

## Introduction

Acrylamide (C3H5ON, CAS79-0601) is a thermal process contaminant that is formed when starchy foods, such as potatoes and cereal products are fried, roasted, or baked at temperatures higher than 120 °C. It is a result of the chemical reactions between a series of certain amino acids (e.g. asparagine) and compounds with carbonyl groups (e.g. glucose, fructose, and maltose). This reaction, known as the Maillard reaction, gives to foods, on one hand, the corresponding color and flavor, and on the other hand, leads to the formation of the undesired acrylamide compound (1, 2). Acrylamide has been classified as ‘probably carcinogenic for humans’(category 2A) (3), a genotoxic (4), mutagen (category 1B) and reproductive toxicant (category 2, fertility) (5). A new EU regulation on acrylamide has set benchmark values in several categories that is 50 μg/kg in Wheat based bread (6). 

In recent years, at the international level, numerous methods for quantification of acrylamide in food have been developed. The most used analytical methods for acrylamide analysis are HPLC-MS/MS and GC-MS (7). Determination of acrylamide by HPLC-MS (/MS) is simpler because acrylamide is determined directly without derivatization, but it requires more sample cleanup prior to analysis to prevent the interference and enhance sensitivity (7–9).

As acrylamide is a small, polar, and hydrophilic molecule, a large amount of matrix interferences tends to be extracted simultaneously with acrylamide, which makes it difficult for analysis (10). Many factors can affect acrylamide extraction yield from food matrices such as sample particle size, extraction solvent, defatting, solvent-to-sample ratio, Ultra Turrax homogenization, the application of mechanical forces, extraction temperature, and extraction time (7). 

For the cleanup of acrylamide from complex samples, accelerated solvent extraction (ASE), liquid-liquid extraction (LLE) and solid-phase extraction (SPE), alone or in combination with other purification steps, were reported by many laboratories. ASE and LLE with conventional organic solvent are time-consuming and labor-intensive. Furthermore, they can easily lead to loss of acrylamide in the preparation process and require large amounts of toxic solvents (10, 11). SPE is also time-consuming, labor-intensive and expensive.

QuEChERS (Quick, Easy, Cheap, Effective, Rugged, and Safe) extraction methods have applied to acrylamide analysis (11). Our objective was to modify QuEChERS sample preparation method and reduce adverse matrix-related effects by constructing spiked calibration curves.

## Experimental

*Sample collection*

Fourteen semi-industrial and twelve traditional bread samples were collected between July 2012 to February 2014 from Sangak bakeries located in Shiraz city (Southwest Iran). All bread samples were baked from wheat flour. After collection, all samples were covered with aluminum foil in order to prevent photodegradation and transported to the laboratory. Each sample (the whole bread) was cut into small pieces and blended. After mixing, the samples were stored in amber glass bottles with Teflon®-lined caps at −20 °C until analysis. 

*Chemicals*

Acrylamide and acrylamide-d_3_ as internal standard (ISTD) were purchased from Sigma Aldrich (St. Louis, Mo., USA). HPLC grade solvents including acetonitrile, acetone and methanol were purchased from Merck (Darmstadt, Germany). Potassium hexacyanoferrate and zinc sulfate were obtained from Chem Lab NV (Belgium) and primary secondary amine (PSA) SPE bulk sorbent purchased from Varian (Italy). Ultrapure water was prepared using an Econolab water purification system (Oklahama, USA). 

*Preparation of standards and reagents*

Acrylamide and acrylamide-d_3_ stock solutions were prepared at 1 mg/mL concen-tration in distilled water. Intermediate standard solutions of acrylamide (100,000 and 10,000 ng/mL) and acrylamide-d_3_ (10,000 ng/mL) were made in distilled water, respectively. Fifty µL of each working standard solution) 20 - 3000 ng/mL ( and 100 μL of acrylamide-d_3_ solution in water (10,000 ng/mL) were added to 1 g of blank bread samples to make the final concentrations of 1, 2.5, 5, 10, 30, 100, and 150 ng/g of acrylamide in bread. For finding blank bread sample, different types of the bread samples that were purchased from different bakeries, located in Shiraz and Tehran cities, were analyzed, and the blank samples were used for validation experiments. 

To avoid light exposure, all standard solutions were prepared in an amber colored volumetric flask and stored at 4 °C until required. The samples so obtained were treated as described in the sample preparation section. Carrez I solution was prepared by dissolving 1.5 g of potassium hexacyanoferrate in 10 mL distilled water , and Carrez II solution prepared by dissolving 3 g of zinc sulfate in 10 mL distilled water.

*Sample preparation*

 The extraction procedure is as follows: 1 g sample was weighed into a 15 mL centrifuge tube, and 100 μL of 10,000 ng/mL of the acrylamide-d_3_ solution and 2.5 mL methanol were added. The tube was shaken by a vortex shaker for 20 s and then the mixture was centrifuged at 4500 RPM for 10 min. The whole methanol extract was transferred to a 15 mL centrifuge tube and then 50 µL of Carrez Ӏ and ӀӀ solutions was added to the tube. The tube was shaken by vortex shaker for 10 s. Fifty mg PSA was added to the tube and then shaken for 10 s. Then, the mixture was centrifuged at 4500 RPM for 10 min. The whole methanol extract was transferred to a 2 mL microtube. The extract was evaporated under gentle flow of nitrogen gas until about 100-150 µL of the extract remained. The remaining extract was dissolved in 500 µL of distilled water and then shaken for 10 s. Finally, 400 µL of the extract was transferred to an amber vial and 70 µL of it was injected to LC-MS/MS.

*Liquid Chromatography—Mass Spectrometry Condition*

The quantification of acrylamide was performed with an Agilent 1200 model HPLC system (Agilent Santa Clara, CA, USA) consisting a binary pump, an autosampler, and a temperature controlled column oven, coupled to an Agilent 6410 Triple Quadrupole mass spectrometer system equipped with electrospray ionization (ESI) interface.

Analytical separation was performed on an ODS-H optimal-C18, Capital (150 mm×4.6 mm, 3 µm) column using an isocratic mixture of 0.1% formic acid in an aqueous solution and 3% methanol (97:3, v/v) at a flow rate of 0.5 mL/min. 

The electrospray was operated in the positive ion mode with a capillary set at 4.0 kV and collision energy at 10 eV. The source gas temperature was set at 325 °C and the desolvation temperature at 400 °C. Nitrogen was used as nebulizer gas (flow 10 l min^−1^), desolvation gas (flow 150 L h^−1^), and collision gas at a pressure of 2.3e^−3^ mbar. Multiple reaction monitoring (MRM) mode of fragmentation patterns m/z 72 → 55 (acrylamide) and m/z 75 → 58 (acrylamide-d_3_) were used for quantitation. 

*Method validation*

For method validation, the parameters assessed were linearity, limit of detection (LOD), limit of quantification (LOQ), recovery, precision, and measurement uncertainty. 

For construction of spiked calibration curve, the spiked bread samples at concentrations of 1, 2.5, 5, 10, 30, 100 and 150 ng/g were prepared in triplicates at three days and then treated according to the procedure described previously. Recoveries were calculated for spiked samples at three levels (1.5, 50 and 130 ng/g) using the spiked calibration curves.

## Results

In method optimization, the following parameters were evaluated: effect of solvent evaporation, effect of simultaneous adding of Carrez and PSA solutions, the effect of adding different amounts of Carrez 1 and 2 solutions and the effect of adding different amounts of PSA.

*Method optimization*

*Effect of adding Carrez and PSA solutions simultaneously*

At this stage, the effect of adding Carrez 1 and 2 and PSA solutions were evaluated separately and simultaneously. For this purpose 70 µL of these solutions (method 1 and 2 explained below) was injected.

1. After adding the Carrez 1 and 2 solutions, the sample was mixed and then PSA was added and after mixing, the sample was centrifuged and eventually other steps of extraction and purification were performed.

2. The samples after the addition of Carrez 1 and 2 solutions were mixed, using vortex mixer and then centrifuged and PSA was added to the supernatant (extraction solution). Other procedures of extraction and purification were performed.

**Figure 1:**
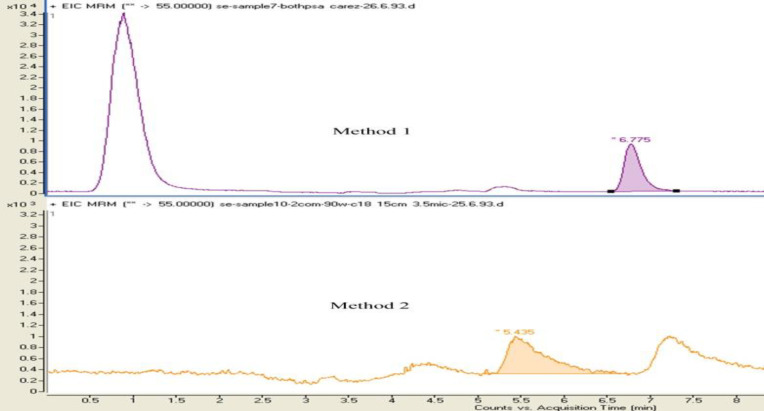
Chromatograms obtained from samples spiked at 150 ng/g of acrylamide when adding Carrez and PSA simultaneously (Method 1) and separately (Method 2).

In the first procedure, in addition to achieving good peak shape) because of removing matrix interferences and clarifying of extract simultaneously (, the centrifuge of this step was removed (Figure 1). 

*The effect of adding different amounts of Carrez 1 and 2 solutions*

**Figure 2:**
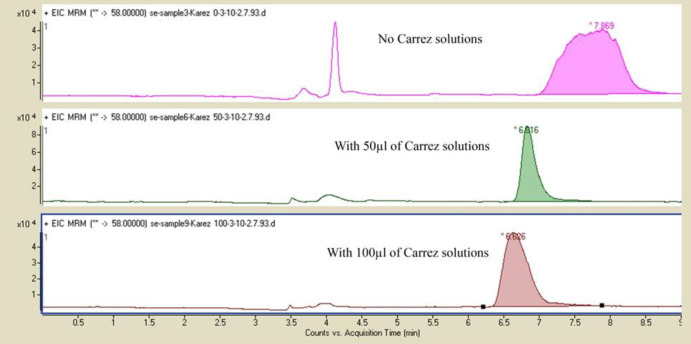
Chromatograms obtained from samples spiked at 50 ng/g of acrylamide without Carrez 1 and 2 solutions and with adding Carrez 1 ansd 2 solutions

At this step, the effect of adding 50 µL of Carrez 1 and 2 solutions and 100 µL Carrez 1 and 2 solutions were evaluated. The results showed good peak shape and repeatability of AUC of peaks due to higher clarification of extract with 50 µL Carrez 1 and 2 solutions on samples spiked at 50 ng /g of acrylamide (Figure 2). 

*The effect of adding different amounts of PSA*

**Figure 3:**
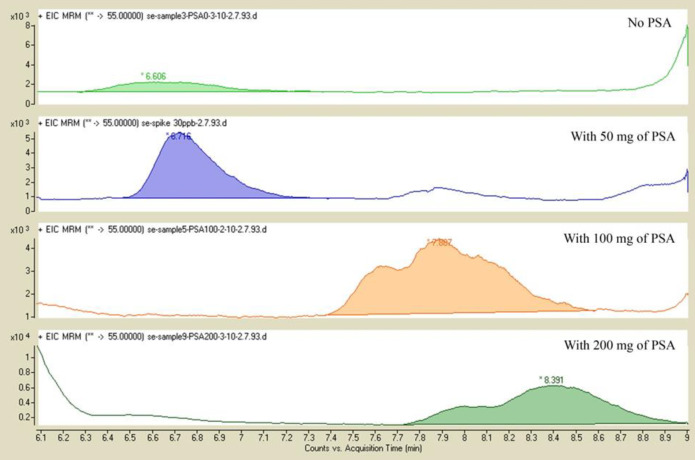
Chromatograms obtained from samples spiked at 50 ng/g of acrylamide without PSA addition, and addition of PSA (50, 100 and 200 mg).

In this part, the effect of addition of PSA at 50, 100, and 200 mg was evaluated. In the absence of PSA, the sample was not very well purified. When 100 and 200 mg PSA was added, the shape of peak and AUC reproducibility was not improved. When 50 mg PSA was added to the extract, matrix co-extractives were more removed and the shape of peak and reproducibility of AUC peaks were improved (Figure 3).

*Method validation*

**Figure 4:**
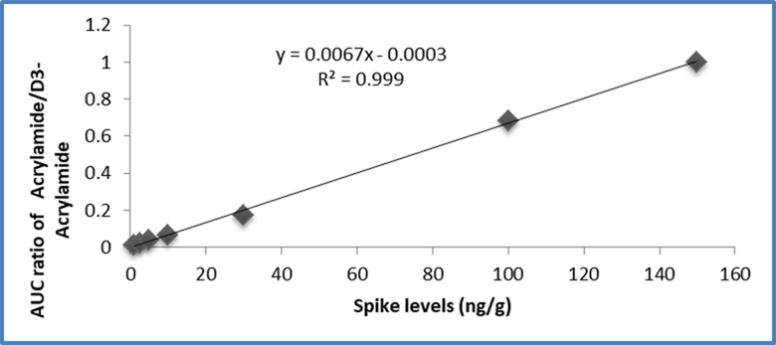
Acrylamide spiked calibration curves in bread samples

*Linearity. *Spiked calibration standards at 1, 2.5, 5, 10, 30, 100, and 150 ng/g were prepared by the addition of 50 μL of 20, 50, 100, 200, 600, 2000, and 3000 ng/mL standard stock solutions to 1 g of the blank bread samples. Quantification of acrylamide in the bread samples was performed using an internal standard method. Calibration curves showed a linear relationship between the concentration and peak area ratios, with a correlation coefficient of 0.999. Figure 4 shows a calibration curve of acrylamide quantification.

**Figure 5:**
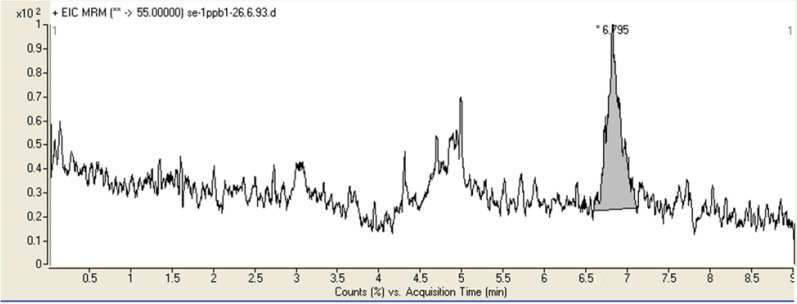
Chromatograms obtained from blank bread spiked with acrylamide at 1 ng/g

The LOD and LOQ of method for acrylamide were 0.3 and 1 ng/g, respectively. Figure 5 shows the chromatograms obtained from blank bread spiked at 1 ng/g with acrylamide. 

*Recovery. *The extraction recoveries were determined by applying the full procedure to triplicate samples in three days at three spiking levels of 1.5, 50 and 130 ng/g (n = 9). The extraction recovery was expressed as recovery percentage. Proper recoveries (96.0-105.3%) of acrylamide from spiked samples were obtained. The values of recovery percentage are presented in Table 1.

*Precision.* The values obtained for CV% were less than 10.8 %. Table 1 shows the calculated CV% at each spiking level.

*Uncertainty. *The expanded measurement uncertainty was calculated using a coverage factor of 2 which gives a level of confidence of approximately 95% (U = 2u) (12). Average Uncertainty of the analytical method was about 20%.

*Application of the optimized method-analysis of unknown bread samples*

To establish the capability and suitability of the developed method, a variety of bread samples were analyzed for the presence of acrylamide. The results of determination of acrylamide in bread samples are presented in Table 2. The mean acrylamide concentration in semi-industrial and traditional Sangak samples was 49 and 42 ng/g, respectively. The results indicate that 100% of the semi-industrial Sangak samples, and 88.9% of the traditional Sangak bread samples were contaminated with acrylamide that 64.3 and 33.3 of semi-industrial and traditional Sangak bread were higher than benchmark levels (50 µg/kg), respectively (6). 

Considering the few Sangak samples analyzed in this study, a comprehensive survey for acrylamide in Sangak bread samples seems to be needed. 

## Discussion

Low molecular weight, high reactivity, and lack of chromophore are challenges in the analysis of acrylamide at low concentrations in food products (13). Accurate quantification of acrylamide in foods by using modern techniques of chromatography and electrophoresis requires complete and laborious isolation of this analyte and purification from co-extractable substances, which can interfere with its assay. In view of the complex character of food matrices, isolation of individual substances is extremely difficult for the analyst (14–16). 

According to the similarity miscibility theory, polar media is used to extract acrylamide, including water, solutions (formic acid) and organic solvents such as n-propanol, 2-butanone, acetone and acetonitrile (8, 13). Water can minimize the collection of hydrophobic compounds in foods but other hydrophilic interferences still remain that should be removed afterwards (14). In comparison with water, organic solvents have the advantages of being able to be extracted even without centrifugation and convenience in evaporation. For protein-rich food samples, Carrez reagents ([I] hexacyanoferrate (II) and [II] zinc sulfate), acetone, ethanol, or methanol was used to precipitate and remove proteins (8).

Using methanol as extraction solvent is one of advantages of this method since ethanol does not extract starch and some other polysaccharides, and also was used to precipitate and remove proteins, so it yields a much clearer extract than that with water even without centrifugation. In addition, it can be easily evaporated under a gentle stream of nitrogen to improve the (LOQ) by concentration (17).

The purification of SPE cartridges was necessary to guarantee the high selectivity of standard methods, but it would increase the cost and complexity of operations (8). 

In this validated method, the sorbent bed was replaced by PSA to which the solution of the sample was added. Techniques based on DSPE permit minimization of additional steps such as precipitation, centrifugation, and filtration, which decreases the manipulation and multi-stage preparation of the sample.

 Mastovska and Lehotay in 2006 used extraction and clean up method with elements from the QuEChERS, the method was directly compatible with both LC-MS and GC-MS techniques (18), but the volume of solvent utilization, time- and labor steps, and material used for extraction is much more than of this method. Table 3 shows examples of analytical methods which have been used in the quantitative analysis of acrylamide in foodstuff.

This protocol of sample preparation in contrast to other methods (Table 3) requires only small volumes of organic solvent. Ease of performance, being inexpensive, and very sensitive are other advantages of this method. Despite the abovementioned advantages, with DSPE technique we could achieve the lowest LOD and LOQ, with the additional advantage of low consumption of solvents in the treatment of the sample. Therefore, it is considered to be a low-cost technique in comparison with classical techniques such as LLE and SPE.

Another advantage of this method is the use of internal standard and construction of a calibration curve by spiked bread samples with acrylamide and acrylamide-d_3_. The addition of an internal standard in food samples and using of spiked calibration curve, preserve possible losses of acrylamide during the sample preparation and overcome the effect of the matrix (32), thus improves the accuracy, precision, and repeatability of measurements. Indeed, in constructing calibration curve by spiked samples, due to similarity of matric compositions in standard sample and unknown samples, the effect of matrix is reﬂected in both standards and unknown samples and also calculation of the analyte (s) concentration in unknown sample is simple without concerning about the matrix effects (33). 

\

**Table 1:** Acrylamide recoveries ± CV (%) and uncertainty (%) for homogenized bread samples using the optimized method at three spiking levels (n = 9)

**Spiking level (ng/g)**	**Average of Recovery (%)**	**CV (%)**	**Uncertainty (%)**
**1.5**	101.2	9.8	19.6
**50**	96.0	10.3	20.6
**130**	105.3	10.8	21.6

**Table 2:** Occurrence of acrylamide in bread samples

**Location**	**Sample**	**Number of samples**	**Number of samples in the range ** **)** **ng/g** **(**		**Mean acrylamide concentration (ng/g)**	**Percent of samples above benchmark levels**
			**<1**	**> 1**		
**Shiraz**	Semi-industrial Sangak bread	14	0	14	49	64.3
	Traditional Sangak bread	12	1	11	42	33.3

**Table 3:** Comparison between this method and other analytical methods which have been used in the quantitative analysis of acrylamide in foodstuff

**Row**	**Matrix**	**LOD (ppb)**	**LOQ (ppb)**	**DLR** ^a^ **(ppb)**	**ISTD** ^b^	**Extraction and clean up method**	**The Method of concentration**	**r** ^2^	**Recovery** **(%)**	**R.S.D.%**	**Extraction solvent**	**Analytical instrument**	**Ref.**
1	Potato chips, biscuits and coffee	2	6	10-1000	AA-d_3_	SPE	Water bath at 40 ◦C and nitrogen	-	92.8-101.5	<4.1	5 mL Methanol	LC-MS/MS	(18)
2	Chicken samples, potato chips, coffee and biscuit	0.5	5	5–100	-	SPE	-	0.99	At least 90%	-	9.8 mL of 0.2 mM acetic acid solutions	LC-MS	(20)
3	Certified reference test material (potato crisps)	1	3	1-200	AA-d_3_	LLE and SPE	-	0.999	81.6–99.0	0.6 to 4.5	> 10 mL Sodium chloride and ethyl acetate	LC-MS/MS	(21)
4	Several typical foods in Spain such as: Christmas sweets, olives, …	2	6	6 ppb to 4ppm	AA-d_3_	SPE	Stream of nitrogen	0.999	-	<13	10 mL Water	LC–MS/MS	(22)
5	Potato crisps and chips, biscuits, crisp breads, pastry, dried fruits, chocolates and coffee	45	100	100 ppb-20ppm	AA-d_3_	SPE	Stream of nitrogen		>85	3–14	10 mL Water	LC–MS/MS	(23)
6	Chips, fries, crisps, breads, biscuits and cookies	6	18	100- 1000	AA-d_3_	SPE	-	0.999	99.7	1.8	10 mL of 0.01 mM acetic acid	LC-MS	(24)
7	Bread	-	25	25-1000	-	DI-SPME	-	0.998	-	-	>10 mL Water	GC–FID	(25)
8	Potato Chips	2.46	3.14	20-400	-	SPE	Vacuum	0.999	84.53-98.37	3-3.74	20 mL of acetone and 100 µL of water	HPLC-UV	(26)
9	Potato crisps and corn products	<10	<30	50–1000	AA-d_3_	Ultrasonic treatment and Carrez solutions	-	-	-	-	20 mL of water	LC–MS/MS	(27)
10	Bread	1.5	5	5-1600	AA-d_3_	SPE with syringe filter	-	0.999	98-101.5	3.08-6.27	5 mL of water	LC–MS/MS	(28)
11	Coffee	-	50	50-1500	2,3,3–d3 AA	SPE	Vacuum	≥0.998	97.4	9.2	30 mL of water	LC–MS/MS	(29)
12	Riceporridge, apple juice and peanut butter	2.69-3.07	8.89-10.13	1-400	AA-d_3_	SPE	-	> 0.995	66.0~118.9	< 20%	9 mL of water	LC–MS/MS	(30)
13	Potato chips and coffee	-	35 (coffee) and 20 (potato chips)	0-1000	-	dispersive SPE	-	0.995-0.999	85-112	5.8-7.6	> 17 mL of n-hexane, water and acetonitrile	LC–MS/MS	(31)
14	Baby food	10	20	10-300	MethAA	SPE	Stream of nitrogen	0.9957	94–110	≤ 10%	10 mL acetonitrile	LC–MS/MS	(32)
15	Bread	0.3	1	1-150	AA-d_3_	Carrez and PSA	Stream of nitrogen	0.999	100.85	10.67	2.5 mL methanol	LC–MS/MS	The proposed method

^a ^dynamic linear range

^b ^Internal standard

## Conclusion

This is a sensitive, cheap and rapid method for preparation of samples and analysis of acrylamide in bread samples. This extraction and clean up method has several advantages, including higher sample throughput and lower costs. It avoids time- and labor-intensive steps such as filtration, multiple SPE cleanups using traditional cartridges. Also, potential contamination by acrylamide from labware is minimized due to the elimination of filters and the use of acrylamide-d_3_ as internal standard significantly improves the accuracy and precision of measurements, allowing repeatability and intermediate reproducibility relative standard deviations below 10%, even at low concentration levels. The use of spiked calibration curves for constructing the calibration curve substantially reduced adverse matrix-related effects. 

The preliminary results showed that the Sangak bread samples could be contaminated with acrylamide and therefore routine monitoring of acrylamide in Sangak bread samples seems necessary.
